# *Erwinia tasmaniensis* levansucrase shows enantiomer selection for (*S*)-1,2,4-butanetriol

**DOI:** 10.1107/S2053230X2200680X

**Published:** 2022-07-26

**Authors:** Ivan Polsinelli, Marco Salomone-Stagni, Stefano Benini

**Affiliations:** a Bioorganic Chemistry and Bio-Crystallography Laboratory (B2Cl), Faculty of Science and Technology, Free University of Bolzano, Piazza Universita 5, 39100 Bolzano, Italy; Baylor College of Medicine, Houston, USA

**Keywords:** fructosyltransferases, transfructosylation, glycosyl hydrolases, microscale thermophoresis, binding assays, levansucrases, *Erwinia tasmaniensis*

## Abstract

Levansucrases are biotechnologically interesting fructosyltransferases due to their potential use in the enzymatic or chemo-enzymatic synthesis of glycosides of non-natural substrates relevant to pharmaceutical applications. The structure of *Erwinia tasmaniensis* levansucrase in complex with (*S*)-1,2,4-butanetriol and its biochemical characterization suggests the possible application of short aliphatic moieties containing polyols with defined stereocentres in fructosylation biotechnology. The structural information revealed that (*S*)-1,2,4-butanetriol mimics the natural substrate. The preference of the protein towards a specific 1,2,4-butanetriol enantiomer was assessed using microscale thermophoresis binding assays. Furthermore, the results obtained and the structural comparison of levansucrases and inulosucrases suggest that the fructose binding modes could differ in fructosyltransferases from Gram-positive and Gram-negative bacteria.

## Abstract 2

Levansucrases are useful tools in biotechnology for the synthesis of fructosyl glycosides. The structure of *Erwinia tasmaniensis* levansucrase in complex with (*S*)-1,2,4-butanetriol suggests a possible influence of polyols with defined stereocentres in the modulation of fructosylation.

## 1. Introduction

Levansucrases (LSCs; EC 2.4.1.10) are members of glycosyl hydrolase family 68 (GH68; Cantarel *et al.*, 2009 ▸). They catalyse either the hydrolysis of sucrose into glucose and fructose or the transfructosylation of a variety of acceptor molecules, forming β-(2,6)-linked oligofructans. These oligosaccharides have a well known prebiotic activity and a wide range of applications (Öner *et al.*, 2016 ▸; Combie & Öner, 2018 ▸; Xu *et al.*, 2019 ▸; González-Garcinuño *et al.*, 2018 ▸). Inulosucrases (INUs; EC 2.4.1.9) belong to the same family and share structural features with LSCs (Pijning *et al.*, 2011 ▸) and perform similar reactions, instead forming β-(2,1)-linked oligofructans (van Hijum *et al.*, 2006 ▸).

LSCs have potential biotechnological applications in the transfructosylation of nonconventional acceptors (Li *et al.*, 2015 ▸). The transglycosylation of molecules may improve their physical, chemical and bioactivity properties (solubility, stability, bioavailability and activity). Enzymatic glycosylation can efficiently produce glycosides, simplifying their *in vitro* synthesis. As an example, the water solubility of phlorizin (a plant metabolite with relevant pharmacological properties), and consequently its bioavailability, was enhanced via fructosylation catalyzed by *Gluconacetobacter diazotrophicus* LSC (Herrera-González *et al.*, 2021 ▸). Phenol derivatives, such as hydroquinone and puerarin, can also be transfructosylated by LSCs from *Bacillus subtilis* (BsSacB) and *G. diazotrophicus* (Mena-Arizmendi *et al.*, 2011 ▸; Núñez-López *et al.*, 2019 ▸). LSCs share the same reaction mechanism (Ortiz-Soto *et al.*, 2019 ▸; Bissaro *et al.*, 2015 ▸) and their active sites have similar structural features (Martínez-Fleites *et al.*, 2005 ▸): a triad of amino acids are involved in catalysis, namely two aspartates and one glutamate (Asp46, Asp203 and Glu287 in *Erwinia tasmani­ensis* LSC and the same residues in *E. amylovora* LSC). While the inner binding site is conserved, surface areas and pocket volumes vary across species due to variability in the surrounding loops (Wuerges *et al.*, 2015 ▸; Ortiz-Soto *et al.*, 2019 ▸).

LSCs from Gram-negative bacteria have successfully been crystallized in complex with sugars in the active site: the structure of *E. amylovora* LSC (EaLsc; Wuerges *et al.*, 2015 ▸; PDB entry 4d47) has been obtained in complex with the sucrose hydrolysis products fructose and glucose, and that of *Beijerinckia indica* LSC (Tonozuka *et al.*, 2020 ▸; PDB entry 6m0e) has been obtained in complex with fructose. LSCs from Gram-positive bacteria have been co-crystallized with fructose (Tonozuka *et al.*, 2012 ▸; PDB entry 3vss), sucrose (Meng & Fütterer, 2003 ▸; PDB entry 1pt2) and raffinose (PDB entry 3byn; G. Meng & K. Fütterer, unpublished work) and also with oligosaccharides with a degree of polymerization up to 6 (PDB entry 6vhq). Inulosucrase from *Lactobacillus johnsonii* (LjInuJ) has been co-crystallized with sucrose and 1-kestose (Pijning *et al.*, 2011 ▸; PDB entries 2yfs and 2yft). Recently, the crystal structure of a fructansucrase from a halophilic archaeal organism, namely the inulin-synthesizing InuHj from *Halalkali­coccus jeotgali* B3T, has been determined in the presence of sucrose and 1-kestose (Ghauri *et al.*, 2021 ▸; PDB entries 7bjc and 7bj4).

Here, we report the crystal structure of *E. tasmaniensis* levansucrase (EtLsc) in complex with (*S*)-1,2,4-butanetriol, which was selected by the enzyme during crystallization from racemic 1,2,4-butanetriol. The preference of the enzyme for the (*S*)-enantiomer was confirmed by microscale thermophoresis binding assays. We analysed the interactions of (*S*)-1,2,4-butanetriol with conserved amino acids in the active site of EtLsc. We compared the binding mode of (*S*)-1,2,4-butanetriol with the binding mode of the fructose moiety found in bacterial LSC and INU structures deposited in the PDB. We propose that the fructose binding modes could differ in Gram-positive and Gram-negative bacteria.

## 2. Materials and methods

### 2.1. Expression and purification of recombinant EtLsc

The production of recombinant levansucrase from *E. tasmaniensis* (strain Et1/99) has previously been described (Polsinelli *et al.*, 2019 ▸). In brief, the levansucrase gene *lsc* was amplified from *E. tasmaniensis* Et1/99 and the PCR product was cloned into pMCSG49 vector (Eschenfeldt *et al.*, 2009 ▸). The protein was expressed in *Escherichia coli* BL21 (DE3) Star pLysS cells. The protein was purified by immobilized metal-affinity chromatography (IMAC), and the His_6_ tag was cleaved with recombinant Tobacco etch virus protease at 4°C overnight and then removed by IMAC. After size-exclusion chromatography the purified EtLsc was concentrated to 10 mg ml^−1^ (in 20 m*M* HEPES pH 7.5, 150 m*M* NaCl) and used for crystallization (Polsinelli *et al.*, 2019 ▸). The protein concentration was assessed using the *A*_280_ method in 6 *M* urea (molecular weight 46 429.9 Da, ɛ_c_ = 84 800 *M* ^−1^ cm^−1^).

### 2.2. Crystallization and X-ray data collection

Crystallization trials were performed using microbatch under oil in 96-well plates with commercial screens, followed by optimization using the hanging-drop vapour-diffusion technique. The structure was obtained using X-ray diffraction data collected from a crystal which grew in drops consisting of 1 µl 12 mg ml^−1^ protein solution (20 m*M* HEPES pH 7.5, 150 m*M* NaCl) and 1 µl crystallization reagent [15% PEG 3000, 20% 1,2,4-butanetriol (CAS 3068-00-6), 1% NDSB 256, 2.5 m*M* manganese(II) chloride tetrahydrate, 2.5 m*M* cobalt(II) chloride hexahydrate, 2.5 m*M* nickel(II) chloride hexahydrate, 2.5 m*M* zinc acetate dihydrate]. Diffraction data were collected on the XRD1 beamline (Lausi *et al.*, 2015 ▸) at the Elettra synchrotron, Trieste, Italy and were processed with *XDS* (Kabsch, 2010 ▸).

The structure was solved by molecular replacement with *MOLREP* (Vagin & Teplyakov, 2010 ▸) using the EaLsc structure (Wuerges *et al.*, 2015 ▸; PDB entry 4d47) as the input model. The model obtained was iteratively refined with *Coot* (Emsley *et al.*, 2010 ▸), *REFMAC*5 (Murshudov *et al.*, 2011 ▸) and *Phenix* (Liebschner *et al.*, 2019 ▸). The *eLBOW* package (Moriarty *et al.*, 2009 ▸) was used to generate the geometry-restraint information for (*S*)-1,2,4-butanetriol. The quality of the model was assessed using *MolProbity* (Williams *et al.*, 2018 ▸). Polder OMIT maps (Liebschner *et al.*, 2017 ▸) were calculated using *Phenix*. Crystallographic figures were created using *PyMOL* (version 2.40; Schrödinger).

### 2.3. Microscale thermophoresis (MST) binding assays

Molecular interactions between EtLsc and (*S*)-1,2,4-butanetriol (CAS 42890-76-6) or (*R*)-1,2,4-butanetriol (CAS 70005-88-8) (both purchased from Sigma–Aldrich) were studied using MST. Ligands were solubilized in MST buffer [20 m*M* HEPES, 150 m*M* NaCl with 0.1%(*v*/*v*) Pluronic F-127] and centrifuged at 10 000*g* for 2 min at 4°C. For the MST assays, a constant concentration of EtLsc in the region of 200 n*M* was titrated with increasing concentrations of ligand. The mixtures were incubated for 30 min at room temperature and then loaded into Monolith NT.LabelFree Capillaries (NanoTemper). Thermophoresis analyses were then performed with Monolith NT.LabelFree (NanoTemper) at low MST power and an LED excitation power of 20%. The dissociation constant values were determined by the NanoTemper analysis software.

## 3. Results and discussion

The structure of EtLsc in complex with (*S*)-1,2,4-butanetriol was solved to a resolution of 1.40 Å (space group *P*4_1_2_1_2). Data-collection and structure-refinement statistics are summarized in Table 1 ▸. Atomic coordinates and experimental structure factors were deposited in the PDB as entry 7oso.

The overall structure is comparable with other EtLsc structures available in the wwPDB (PDB entries 6frw and 6rv5; Polsinelli *et al.*, 2019 ▸, 2020 ▸). No key differences are evident, as confirmed by C^α^ r.m.s.d. values of 0.483 and 0.562 Å, respectively.

GH68 family members perform hydrolysis and transfructosylation through a double-displacement reaction (Bissaro *et al.*, 2015 ▸; Ortiz-Soto *et al.*, 2019 ▸; Homann *et al.*, 2007 ▸). It involves hydrolysis of the glycosidic bond in a fructosyl donor. The glucose moiety is then released, while a fructosyl-enzyme intermediate coordinated by the catalytic triad is formed. Subsequently, the fructosyl moiety is transferred from the enzyme to an acceptor molecule (Raga-Carbajal *et al.*, 2018 ▸).

**Figure 1:**
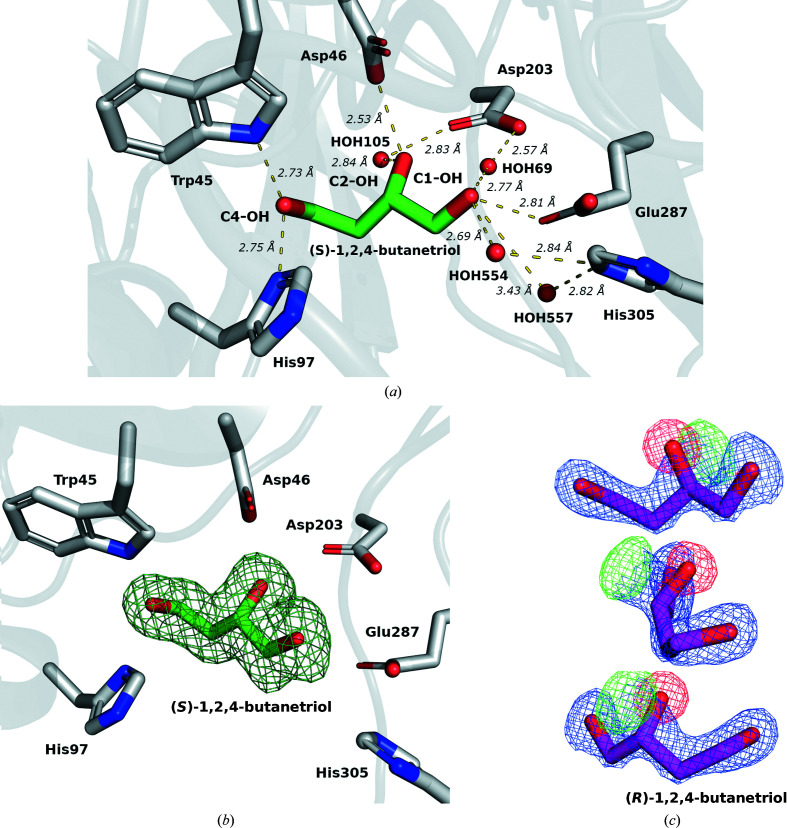
(*a*) Interactions of (*S*)-1,2,4-butanetriol with EtLsc (PDB entry 7oso). The ligand C atoms are coloured green, with EtLsc C atoms in grey, O atoms in red and N atoms in blue. The ligand interacts with the catalytic triad of EtLsc (Asp46, Asp203 and Glu287). (*b*) Representation of a polder OMIT map calculated with exclusion of the (*S*)-1,2,4-butanetriol molecule. The polder map is contoured at 5σ. (*c*) 2*F*_obs_ − *F*_calc_ and *F*_obs_ − *F*_calc_ electron-density maps of (*R*)-1,2,4-butanetriol. The *F*_obs_ − *F*_calc_ map difference-peak intensities are contoured at 10σ.

To give a clearer description, the binding sites of LSCs and INUs are generally divided into subsites associated with their sugar moiety in relation to the bond hydrolysed by the enzyme (site 0). Up to five substrate-binding subsites (−1, +1, +2, +3, and +4) have been described (Raga-Carbajal *et al.*, 2021 ▸).

(*S*)-1,2,4-Butanetriol binds at subsite −1 in the enzyme funnel, where the active-site residues Asp46, Asp203 and Glu287 are located (Fig. 1 ▸ *a*). The compound establishes hydrogen bonds via its OH groups to Asp46 O^δ2^, which binds to C2-OH (2.53 Å), and Glu287 O^ɛ1^, which binds to C1-OH (2.81 Å). C1-OH further binds to HOH69 (2.77 Å), while C2-OH binds to HOH105 (2.84 Å). HOH105 makes a hydrogen bond to Asp203 O^δ1^ (2.83 Å), while HOH69 also bridges to Asp203 O^δ2^ (2.57 Å). C4-OH of (*S*)-1,2,4-butanetriol forms hydrogen bonds to Trp45 N^ɛ1^ (2.73 Å) and His97 N^δ1^ (2.75 Å). The (*S*)-1,2,4-butanetriol OH groups C1-OH and C2-OH establish hydrogen bonds similar to the C3-OH and C4-OH groups of the fructosyl moiety of bound sucrose, which are stabilized by Glu287 (Meng & Fütterer, 2003 ▸; Ozimek *et al.*, 2004 ▸; Rye & Withers, 2000 ▸). Asp46, Asp203 and Glu287 are known to be fundamental for LSC activity.

In *Zymomonas mobilis*, in which two distinct frucosyltransferases are present, a β-fructofuranosidase (ZmFFZm) and a levansucrase (ZmLSZm), His79 of ZmFFZm and Asn84 of ZmLSZm, corresponding to His97 of EtLsc, have been proposed to play a fundamental role in the reaction, acting as the switch from β-(2→1)-transfructosylation to β-(2→6)-transfructosylation (Okuyama *et al.*, 2021 ▸). Furthermore, in the ZmFFZm homology model of Okuyama and coworkers, His79 forms a hydrogen bond to C6-OH of the fructosyl residue at the +1 subsite similar to that observed between C4-OH of (*S*)-1,2,4-butanetriol and His97 of EtLsc.

**Figure 2:**
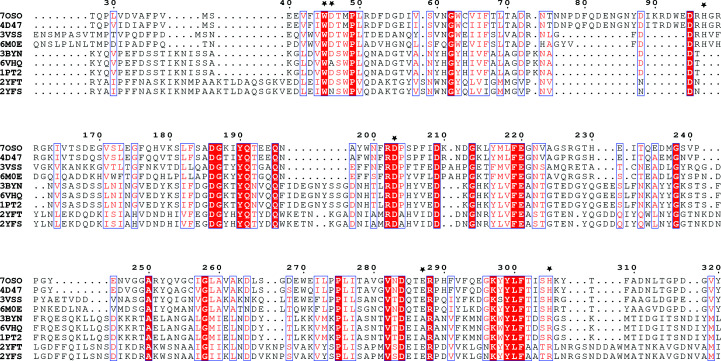
Sequence alignment and residue conservation. Alignment of sequences from the structures analyzed in this paper. EtLsc residues that interact with the ligand are marked with a black star above the alignment. Note that PDB entries 6vhq, 2yfs, 3byn and 1pt2 contain mutations.

Alignment of the sequences from the PDB (Fig. 2 ▸) confirms that the EtLsc residues interacting with (*S*)-1,2,4-butanetriol are conserved. Trp45 and Asp203 are highly conserved, as well as Asp46 and Glu287. His97 is conserved where present (a gap is present in the alignment for BsSacB and LjInuJ). His305 is conserved or substituted with another positively charged amino acid, namely an arginine.

**Figure 3:**
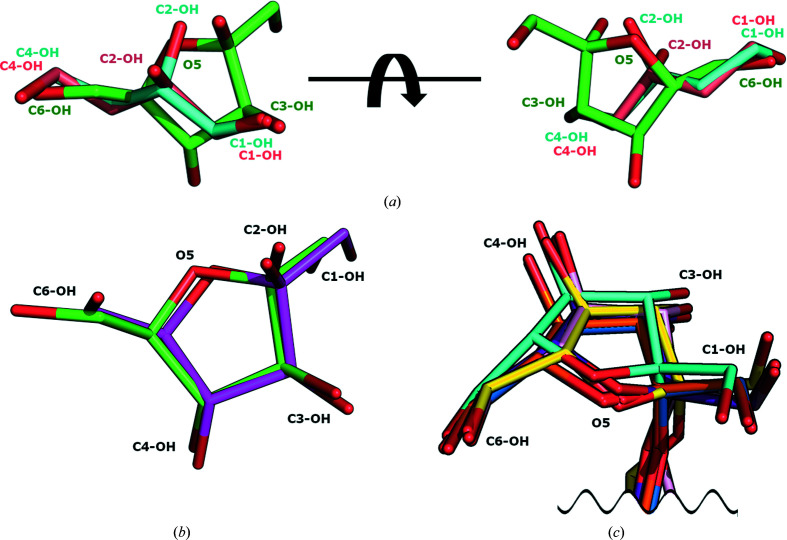
Comparison of the binding modes of (*S*)-1,2,4-butanetriol, (*R*)-1,2,4-butanetriol and fructose. (*a*) (*S*)-1,2,4-butanetriol (PDB entry 7oso; cyan) and (*R*)-1,2,4-butanetriol (salmon) superposed on fructose from EaLsc (PDB entry 4d47; green). (*b*) Fructose binding mode in the Gram-negative EaLsc (PDB entry 4d47; green) and BiBftA (PDB entry 6m0e; magenta). (*c*) Fructose and the fructosyl moiety binding mode in Gram-positive enzymes. Fructose_6_ (PDB entry 6vhq), raffinose (PDB entry 3byn) and sucrose (PDB entry 1pt2) from BsSacB are shown in yellow, blue and orange, respectively. Fructose from ArFFase (PDB entry 3vss) is shown in cyan. Sucrose and 1-kestose from LjInuJ (PDB entries 2yfs and 2yft) are shown in purple and pink, respectively.

Despite the fact that the crystallization conditions included a mixture of the enantiomers (*S*)-1,2,4-butanetriol and (*R*)-1,2,4-butanetriol, the electron-density maps (Polder omit, *F*_obs_ − *F*_calc_ and 2*F*_obs_ − *F*_calc_) show exclusively (*S*)-1,2,4-butanetriol. Binding of the ligand is supported by a Polder map contoured at 5σ (Fig. 1 ▸ *b*; Liebschner *et al.*, 2017 ▸). Fitting the *R* enantiomer gave strong positive and negative peaks in the *F*_obs_ − *F*_calc_ map (contoured at 10σ; Fig. 1 ▸ *c*). Therefore, EtLsc has a lower or no affinity for the ligand with the *R* configuration, as the different stereochemistry at C2 would prevent C2-OH from establishing a hydrogen bond to Asp46 O^δ2^ and give rise to unfavourable interactions with the surrounding residues. The *S* configuration allows the compound to partially mimic the natural substrate, with C2-OH overlapping O5 of fructose (Fig. 3 ▸ *a*), whereas the *R* enantiomer cannot overlap in a similar way in relation to O5. This explains the preference of EtLsc for one of the enantiomers, highlighting the relevance of stereochemistry when screening potential nonstandard acceptors for this class of enzyme.

Although butanol has already been tested as a fructosyl acceptor in BsSacB without a relevant yield (Mena-Arizmendi *et al.*, 2011 ▸), the three OH groups and the chiral centre in (*S*)-1,2,4-butanetriol mimic the fructosyl moiety in the activated complex/second transition state of the reaction. Based on the structural evidence presented here, (*S*)-1,2,4-butanetriol and other chiral polyols are potential candidates as a fructosyl acceptor for BsSacB and other similar LSCs or might modulate or influence transfructosylation.

To compare the binding affinity of EtLsc for (*S*)-1,2,4-butanetriol and (*R*)-1,2,4-butanetriol, both compounds were tested as pure enantiomers by MST. The resulting *K*_d_ for (*S*)-1,2,4-butanetriol is 84.9 ± 13.4 m*M* (Supplementary Fig. S1). It was not possible to measure any affinity for (*R*)-1,2,4-butanetriol in the same concentration range.

### 3.1. Structural comparisons

The EtLsc structure was compared with the structures of similar glycoside hydrolases from family 68 in complex with ligands present in the PDB (Supplementary Fig. S2 and Supplementary Table S1). The structure of EtLsc is very similar to that of EaLsc from *E. amylovora* (Wuerges *et al.*, 2015 ▸; PDB entry 4d47), with a C^α^ r.m.s.d. of 0.396 Å and a sequence identity of 91.6%. The next most similar are BftA from *B. indica* (BiBftA), with an r.m.s.d. of 1.468 Å (Tonozuka *et al.*, 2020 ▸; PDB entry 6m0e), and FFase from *Microbacterium saccharophilum* (ArFFase), formally known as *Arthrobacter* sp. K-1, with an r.m.s.d. of 1.5 Å (Tonozuka *et al.*, 2012 ▸; PDB entry 3vss). The structures of BsSacB from *B. subtilis* show r.m.s.d.s in the range between 1.959 and 1.986 Å (PDB entries 6vhq, 3byn and 1pt2; Meng & Fütterer, 2003 ▸; Raga-Carbajal *et al.*, 2021 ▸). The alignment with the least similarity is with the inulocrase from *L. johnsonii* (LjInuJ), with an r.m.s.d. of 2.165 Å (Pijning *et al.*, 2011 ▸; PDB entries 2yfs and 2yft).

We compared the binding mode of (*S*)-1,2,4-butanetriol with the fructose binding mode in the available structures of bacterial GH68 members. In the Gram-negative GH68 members EaLsc and BiBftA, C1-OH, C2-OH and C4-OH of (*S*)-1,2,4-butanetriol overlap with C3-OH, O5 and C6-OH of fructose, respectively (Fig. 3 ▸ *a*). While it is possible to superimpose (*S*)-1,2,4-butanetriol on the fructose moiety in the Gram-negative bacterial enzymes, it is not possible to do the same in those from Gram-positive bacteria as the fructose moiety binds differently in Gram-negative and Gram-positive bacteria (Figs. 3 ▸ *b* and 3 ▸ *c*).

**Figure 4:**
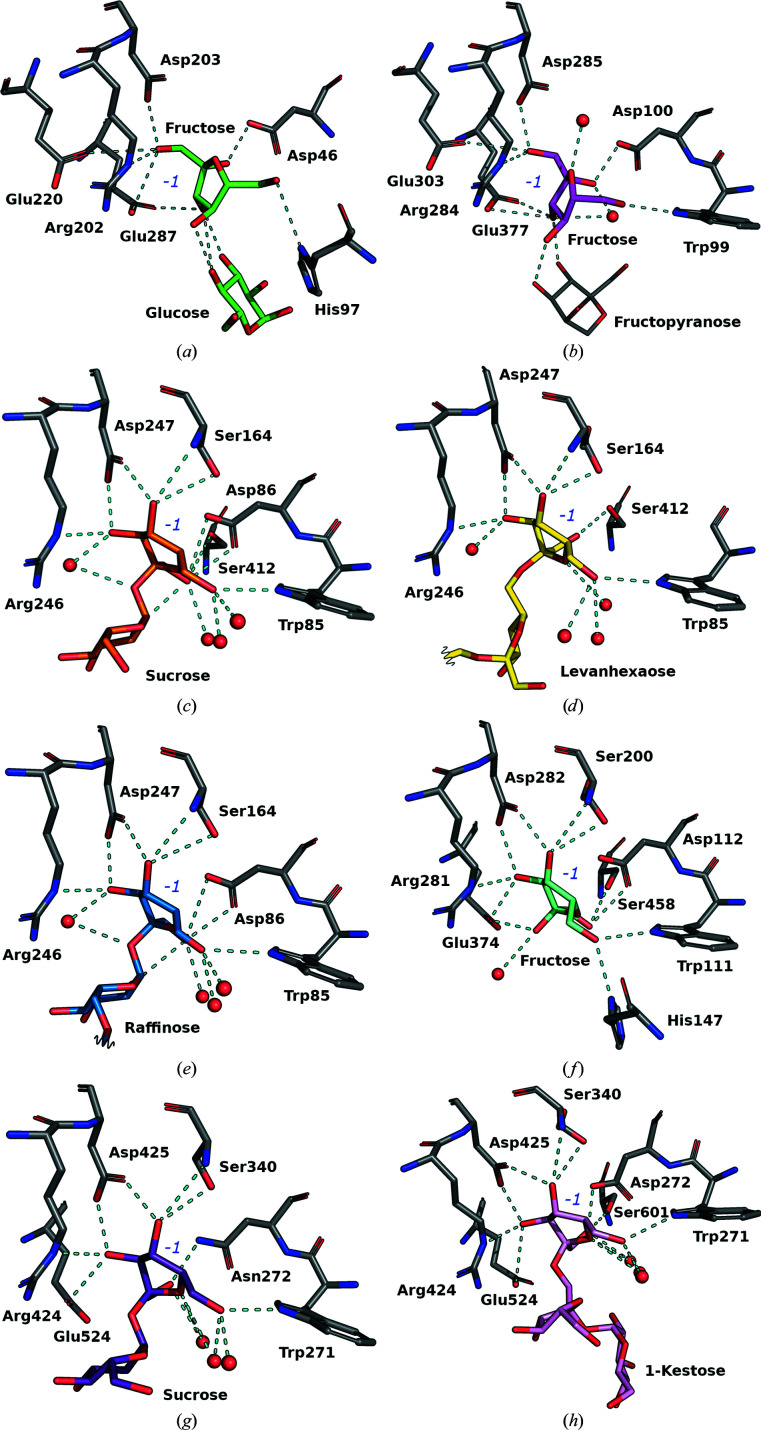
Fructose binding-site comparisons. (*a*) EaLsc (PDB entry 4d47). (*b*) BiBftA (PDB entry 6m0e). (*c*) BsSacB (PDB entry 1pt2). (*d*) BsSacB (PDB entry 6vhq). (*e*) BsSacB (PDB entry 3byn). (*f*) ArFFase (PDB entry 3vss). (*g*) LjInuJ (PDB entry 2yfs) (*h*) LjInuJ (PDB entry 2yft).

All of the LSCs and INUs belong to the same family and have similar structural features, such as the sucrose-binding pocket (Pijning *et al.*, 2011 ▸; Wuerges *et al.*, 2015 ▸). While subsite −1 is highly specific for a fructosyl moiety, other subsites such as subsites +1, +3 and +4 have been shown to be involved in the binding of fructooligosaccharides (FOS; Raga-Carbajal *et al.*, 2021 ▸). Therefore, differences in the acceptor specificity and in the elongation process could be related to the outer part of the pocket. However, the lack of Gram-negative bacterial structures in complex with oligosaccharides in the active site means that this this hypothesis cannot be confirmed. Nevertheless, analysis of the fructose binding network in the available structures (Fig. 4 ▸) highlights some peculiar differences between Gram-negative and Gram-positive bacteria. The fructosyl moiety is oriented differently in Gram-negative (Figs. 4 ▸ *a* and 4 ▸ *b*) and Gram-positive (Figs. 4 ▸ *c*–4 ▸ *h*) bacterial structures, despite the conserved pocket at subsite −1. The C6-OH of fructose in Gram-positive enzymes interacts with His97 in EaLsc and with Trp99 in BiBftA. Similarly, C6-OH of fructose interacts with tryptophan in Gram-positive enzymes (Trp85 in BsSacB, Trp111 in ArFFase and Trp271 in LjInuJ) and also with a histidine in ArFFase (His147).

The C1-OH of fructose in Gram-negative bacterial structures (PDB entry 4d47, Fig. 4 ▸ *a*; PDB entry 6m0e, Fig. 4 ▸ *b*) interacts with glutamic acid (Glu220 and Glu287 in EaLsc and Glu303 and Glu307 in BiBftA), aspartic acid (Asp203 in EaLsc and Asp285 in BiBftA) and arginine (Arg202 in EaLsc and Arg284 in BiBftA), while in Gram-positive bacterial enzymes C1-OH binds to a serine (Ser412 in BsSacB, Ser458 in ArFFase and Ser601 in LjInuJ) and an aspartate (Asp86 in BsSacB, Asp112 in ArFFase and Asp272 in LjInuJ).

Additionally, in the Gram-positive binding pocket a serine (Ser164 in BsSacB, Ser200 in ArFFase and Ser340 in LjInuJ) stabilizes C4-OH of fructose. This serine is either missing in Gram-negative structures (EaLsc and EtLsc have an alanine at this position) or is not involved in fructose binding (as in BiBftA). This serine has been shown to play a role in the BsSacB product profile, as an S164A variant of BsSacB was found to show enhanced production of blasto-FOS (Ortiz-Soto *et al.*, 2020 ▸). Recently, the structure of a fructansucrase from *H. jeotgali* B3T, a halophilic archaeal organism, has been determined. This enzyme is structurally closer to the LSCs from Gram-negative bacteria than to the Gram-positive bacterial LSCs analysed in this work. However, the fructose binding mode of sucrose and 1-kestose (Ghauri *et al.*, 2021 ▸; PDB entries 7bjc and 7bj4) is comparable with those of LSCs and INUs present in Gram-positive bacteria (Supplementary Fig. S3).

Despite the common structural features and similar binding sites of Gram-negative and Gram-positive bacterial LSCs and INUs, there is evidence suggesting that their biochemical behaviour could be related to a few small peculiarities; for example, the fructose binding mode and the presence/absence of the serine that binds fructose in subsite −1. These differences, together with the divergences in other subsites involved in FOS binding (Raga-Carbajal *et al.*, 2021 ▸), might be relevant to understanding the mechanism regulating chain elongation. However, further studies are required to elucidate the reasons for the different fructose binding modes observed in Gram-positive and Gram-negative family GH68 members and the consequences for the product length and the elongation process.

## 4. Conclusions

The transfructosylation of bioactive molecules may lead to glycosides relevant to pharmaceutical applications. Levan­sucrases and inulosucrases are promising tools for transfructosylation and there is a particular interest in discovering new nonstandard acceptors. The structure that we present here contains a polyalcohol moiety with a defined stereochemistry. Although butanol has already been tested as a fructosyl acceptor in BsSacB without a relevant yield (Mena-Arizmendi *et al.*, 2011 ▸), the three OH groups and the chiral centre in (*S*)-1,2,4-butanetriol might mimic the fructosyl moiety in the activated complex/second transition state of the reaction. The structural data, supported by the affinity study, increase the interest in the potential application of EtLsc and other levansucrases and inulosucrases in the fructosylation of non­standard acceptors. In fact, this study suggests that the pocket of EtLsc could select a stereospecific polyalcohol motif, making the enzyme a candidate for testing the transfructosylation of molecules including polyalcohols (such as sugar alcohols) or molecules containing polyol moieties. Mutants could be designed based on these structural data to engineer enzymes with the desired preference for this type of non­standard substrate, for example to synthesize glycosides for pharmaceutical applications. Further studies are required to elucidate the reasons for the different fructose binding modes observed in Gram-positive and Gram-negative family 68 glycoside hydrolases and the consequences for the product length, the implications for the elongation process and the differences in the product spectrum.

## Supplementary Material

PDB reference: *Erwinia tasmaniensis* levansucrase, 7oso

Supplementary Table and Figures. DOI: 10.1107/S2053230X2200680X/ft5120sup1.pdf

## Figures and Tables

**Table 1:** Data-collection and refinement statistics for *E. tasmaniensis* levansucrase (PDB entry 7oso) Values in parentheses are for the outer shell.

Diffraction source	XRD1 beamline, Elettra
Wavelength (Å)	1.00
Temperature (K)	100
Detector	Dectris PILATUS3 S 2M
Space group	*P*4_1_2_1_2
*a*, *b*, *c* (Å)	127.713, 127.713, 61.016
α, β, γ (°)	90, 90, 90
Mosaicity (°)	0.137
Resolution range (Å)	30.63–1.40 (1.45–1.40)
Total No. of reflections	1063787 (166093)
No. of unique reflections	189742 (30398)
Completeness (%)	99.91 (99.53)
Multiplicity	5.6 (5.4)
〈*I*/σ(*I*)〉	23.25 (1.26†)
*R*_meas_	0.0531 (1.886)
*R*_merge_	0.05056 (1.793)
CC_1/2_	1.000 (0.686)
Overall *B* factor from Wilson plot (Å^2^)	20.95
No. of reflections, working set	99146 (9753)
No. of reflections, test set	4935 (470)
Final *R*_work_	0.134
Final *R*_free_	0.164
Cruickshank DPI (Å)	0.044
No. of non-H atoms	
Protein	3349
Ion	3
Ligand	7
Water	596
Total	3955
R.m.s. deviations	
Bond lengths (Å)	0.008
Angles (°)	1.47
Average *B* factors (Å^2^)	
Overall	30.78
Protein	27.8
Ion	28.05
Ligand	24.93
Water	47.32
Ramachandran plot	
Most favoured (%)	96.6
Allowed (%)	3.4

† *I*/σ(*I*) falls below 2.0 at 1.5 Å resolution.

## References

[bb2] Bissaro, B., Monsan, P., Fauré, R. & O’Donohue, M. J. (2015). *Biochem. J.* **467**, 17–35. 10.1042/BJ20141412 25793417

[bb3] Cantarel, B. L., Coutinho, P. M., Rancurel, C., Bernard, T., Lombard, V. & Henrissat, B. (2009). *Nucleic Acids Res.* **37**, D233–D238. 10.1093/nar/gkn663 PMC2686590 18838391

[bb4] Combie, J. & Öner, E. T. (2018). *Bioinspir. Biomim.* **14**, 011001. 10.1088/1748-3190/aaed92 30457113

[bb5] Emsley, P., Lohkamp, B., Scott, W. G. & Cowtan, K. (2010). *Acta Cryst.* D**66**, 486–501. 10.1107/S0907444910007493 PMC2852313 20383002

[bb6] Eschenfeldt, W. H., Lucy, S., Millard, C. S., Joachimiak, A. & Mark, I. D. (2009). *Methods Mol. Biol.* **498**, 105–115. 10.1007/978-1-59745-196-3_7 PMC2771622 18988021

[bb7] Ghauri, K., Pijning, T., Munawar, N., Ali, H., Ghauri, M. A., Anwar, M. A. & Wallis, R. (2021). *FEBS J.* **288**, 5723–5736. 10.1111/febs.15843 33783128

[bb8] González-Garcinuño, Á., Tabernero, A., Domínguez, Á., Galán, M. A. & Martin del Valle, E. M. (2018). *Biocatal. Biotransformation*, **36**, 233–244.

[bb9] Herrera-González, A., Núñez-López, G., Núñez-Dallos, N., Amaya-Delgado, L., Sandoval, G., Remaud-Simeon, M., Morel, S., Arrizon, J. & Hernández, L. (2021). *Enzyme Microb. Technol.* **147**, 109783. 10.1016/j.enzmictec.2021.109783 33992405

[bb10] Hijum, S. A. F. T. van, Kralj, S., Ozimek, L. K., Dijkhuizen, L. & van Geel-Schutten, I. G. (2006). *Microbiol. Mol. Biol. Rev.* **70**, 157–176. 10.1128/MMBR.70.1.157-176.2006 PMC1393251 16524921

[bb11] Homann, A., Biedendieck, R., Götze, S., Jahn, D. & Seibel, J. (2007). *Biochem. J.* **407**, 189–198. 10.1042/BJ20070600 PMC2049016 17608626

[bb12] Kabsch, W. (2010). *Acta Cryst.* D**66**, 125–132. 10.1107/S0907444909047337 PMC2815665 20124692

[bb13] Lausi, A., Polentarutti, M., Onesti, S., Plaisier, J. R., Busetto, E., Bais, G., Barba, L., Cassetta, A., Campi, G., Lamba, D., Pifferi, A., Mande, S. C., Sarma, D. D., Sharma, S. M. & Paolucci, G. (2015). *Eur. Phys. J. Plus*, **130**, 43.

[bb14] Li, W., Yu, S., Zhang, T., Jiang, B. & Mu, W. (2015). *Appl. Microbiol. Biotechnol.* **99**, 6959–6969. 10.1007/s00253-015-6797-5 26160392

[bb1] Liebschner, D., Afonine, P. V., Baker, M. L., Bunkóczi, G., Chen, V. B., Croll, T. I., Hintze, B., Hung, L.-W., Jain, S., McCoy, A. J., Moriarty, N. W., Oeffner, R. D., Poon, B. K., Prisant, M. G., Read, R. J., Richardson, J. S., Richardson, D. C., Sammito, M. D., Sobolev, O. V., Stockwell, D. H., Terwilliger, T. C., Urzhumtsev, A. G., Videau, L. L., Williams, C. J. & Adams, P. D. (2019). *Acta Cryst.* D**75**, 861–877.

[bb15] Liebschner, D., Afonine, P. V., Moriarty, N. W., Poon, B. K., Sobolev, O. V., Terwilliger, T. C. & Adams, P. D. (2017). *Acta Cryst.* D**73**, 148–157. 10.1107/S2059798316018210 PMC5297918 28177311

[bb16] Martínez-Fleites, C., Ortíz-Lombardía, M., Pons, T., Tarbouriech, N., Taylor, E. J., Arrieta, J. G., Hernández, L. & Davies, G. J. (2005). *Biochem. J.* **390**, 19–27. 10.1042/BJ20050324 PMC1188265 15869470

[bb17] Mena-Arizmendi, A., Alderete, J., Águila, S., Marty, A., Miranda-Molina, A., López-Munguía, A. & Castillo, E. (2011). *J. Mol. Catal. B Enzym.* **70**, 41–48.

[bb18] Meng, G. & Fütterer, K. (2003). *Nat. Struct. Mol. Biol.* **10**, 935–941. 10.1038/nsb974 14517548

[bb19] Moriarty, N. W., Grosse-Kunstleve, R. W. & Adams, P. D. (2009). *Acta Cryst.* D**65**, 1074–1080. 10.1107/S0907444909029436 PMC2748967 19770504

[bb20] Murshudov, G. N., Skubák, P., Lebedev, A. A., Pannu, N. S., Steiner, R. A., Nicholls, R. A., Winn, M. D., Long, F. & Vagin, A. A. (2011). *Acta Cryst.* D**67**, 355–367. 10.1107/S0907444911001314 PMC3069751 21460454

[bb21] Núñez-López, G., Herrera-González, A., Hernández, L., Amaya-Delgado, L., Sandoval, G., Gschaedler, A., Arrizon, J., Remaud-Simeon, M. & Morel, S. (2019). *Enzyme Microb. Technol.* **122**, 19–25. 10.1016/j.enzmictec.2018.12.004 30638505

[bb22] Okuyama, M., Serizawa, R., Tanuma, M., Kikuchi, A., Sadahiro, J., Tagami, T., Lang, W. & Kimura, A. (2021). *J. Biol. Chem.* **296**, 100398. 10.1016/j.jbc.2021.100398 PMC7961098 33571525

[bb23] Öner, E. T., Hernández, L. & Combie, J. (2016). *Biotechnol. Adv.* **34**, 827–844. 10.1016/j.biotechadv.2016.05.002 27178733

[bb24] Ortiz-Soto, M. E., Porras-Domínguez, J. R., Rodríguez-Alegría, M. E., Morales-Moreno, L. A., Díaz-Vilchis, A., Rudiño-Piñera, E., Beltrán-Hernandez, N. E., Rivera, H. M., Seibel, J. & López Munguía, A. (2020). *Int. J. Biol. Macromol.* **161**, 898–908. 10.1016/j.ijbiomac.2020.06.114 32553967

[bb25] Ortiz-Soto, M. E., Porras-Domínguez, J. R., Seibel, J. & López-Munguía, A. (2019). *Carbohydr. Polym.* **219**, 130–142. 10.1016/j.carbpol.2019.05.014 31151510

[bb26] Ozimek, L. K., van Hijum, S. A. F. T., van Koningsveld, G. A., van der Maarel, M. J. E. C., van Geel-Schutten, G. H. & Dijkhuizen, L. (2004). *FEBS Lett.* **560**, 131–133. 10.1016/S0014-5793(04)00085-7 14988011

[bb27] Pijning, T., Anwar, M. A., Böger, M., Dobruchowska, J. M., Leemhuis, H., Kralj, S., Dijkhuizen, L. & Dijkstra, B. W. (2011). *J. Mol. Biol.* **412**, 80–93. 10.1016/j.jmb.2011.07.031 21801732

[bb28] Polsinelli, I., Caliandro, R., Demitri, N. & Benini, S. (2020). *Int. J. Mol. Sci.* **21**, 83. 10.3390/ijms21010083 PMC6981717 31877648

[bb29] Polsinelli, I., Caliandro, R., Salomone-Stagni, M., Demitri, N., Rejzek, M., Field, R. A. & Benini, S. (2019). *Int. J. Biol. Macromol.* **127**, 496–501. 10.1016/j.ijbiomac.2019.01.074 30660564

[bb30] Raga-Carbajal, E., Díaz-Vilchis, A., Rojas-Trejo, S. P., Rudiño-Piñera, E. & Olvera, C. (2021). *J. Biol. Chem.* **296**, 100178. 10.1074/jbc.RA120.015853 PMC7948499 33303628

[bb31] Raga-Carbajal, E., López-Munguía, A., Alvarez, L. & Olvera, C. (2018). *Sci. Rep.* **8**, 15035. 10.1038/s41598-018-32872-7 PMC6177408 30301900

[bb32] Rye, C. S. & Withers, S. G. (2000). *Curr. Opin. Chem. Biol.* **4**, 573–580. 10.1016/s1367-5931(00)00135-6 11006547

[bb33] Tonozuka, T., Kitamura, J., Nagaya, M., Kawai, R., Nishikawa, A., Hirano, K., Tamura, K., Fujii, T. & Tochio, T. (2020). *Biosci. Biotechnol. Biochem.* **84**, 2508–2520. 10.1080/09168451.2020.1804317 32752982

[bb34] Tonozuka, T., Tamaki, A., Yokoi, G., Miyazaki, T., Ichikawa, M., Nishikawa, A., Ohta, Y., Hidaka, Y., Katayama, K., Hatada, Y., Ito, T. & Fujita, K. (2012). *Enzyme Microb. Technol.* **51**, 359–365. 10.1016/j.enzmictec.2012.08.004 23040392

[bb35] Vagin, A. & Teplyakov, A. (2010). *Acta Cryst.* D**66**, 22–25. 10.1107/S0907444909042589 20057045

[bb36] Williams, C. J., Headd, J. J., Moriarty, N. W., Prisant, M. G., Videau, L. L., Deis, L. N., Verma, V., Keedy, D. A., Hintze, B. J., Chen, V. B., Jain, S., Lewis, S. M., Arendall, W. B. III, Snoeyink, J., Adams, P. D., Lovell, S. C., Richardson, J. S. & Richardson, D. C. (2018). *Protein Sci.* **27**, 293–315. 10.1002/pro.3330 PMC5734394 29067766

[bb37] Wuerges, J., Caputi, L., Cianci, M., Boivin, S., Meijers, R. & Benini, S. (2015). *J. Struct. Biol.* **191**, 290–298. 10.1016/j.jsb.2015.07.010 26208466

[bb38] Xu, W., Ni, D., Zhang, W., Guang, C., Zhang, T. & Mu, W. (2019). *Crit. Rev. Food Sci. Nutr.* **59**, 3630–3647. 10.1080/10408398.2018.1506421 30595032

