# Supplementary material for: Erwinia tasmaniensis levansucrase shows enantiomer selection for (S)-1,2,4-butanetriol
Source: Acta Crystallogr F Struct Biol Commun. 2022 Jul 26;78(Pt 8):289–96. doi: 10.1107/S2053230X2200680X (PMC9350837; doi:10.1107/S2053230X2200680X)
Supplement: Supplementary file 1 [file f-78-00289-sup1.pdf]

STRUCTURAL BIOLOGY COMMUNICATIONS

Volume 78 (2022)

Supporting information for article:

Erwinia tasmaniensis levansucrase shows enantiomer selection for (*S*)-1,2,4-butanetriol

Ivan Polsinelli, Marco Salomone-Stagni and Stefano Benini

Table S1 Structures and sequence identity comparison of bacterial LSCs and INUs.

RMSD (Å)									
	EtLsc (7OSO)	EaLsc (4D47)	ArFFase (3VSS)	BiBftA (6M0E)	BsSacB (3BYN)	BsSacB (6VHQ)	BsSacB (1PT2)	LjInuJ (2YFT)	LjInuJ (2YFS)
EtLsc (7OSO)		0.396	1.5	1.468	1.959	1.986	1.96	2.165	2.165
EaLsc (4D47)	0.396		1.492	1.484	1.952	1.975	1.952	2.119	2.12
ArFFase (3VSS)	1.5	1.492		0.941	1.828	1.825	1.825	2.075	2.075
BiBftA (6M0E)	1.468	1.484	0.941		1.878	1.898	1.875	2.092	2.095
BsSacB (3BYN)	1.959	1.952	1.828	1.878		0.369	0.085	1.263	1.268
BsSacB (6VHQ)	1.986	1.975	1.825	1.898	0.369		0.368	1.322	1.33
BsSacB (1PT2)	1.96	1.952	1.825	1.875	0.085	0.368		1.261	1.267
LjInuJ (2YFT)	2.165	2.119	2.075	2.092	1.263	1.322	1.261		0.132
LjInuJ (2YFS)	2.165	2.12	2.075	2.095	1.268	1.33	1.267	0.132	

Sequence Identity									
	EtLsc (7OSO)	EaLsc (4D47)	ArFFase (3VSS)	BiBftA (6M0E)	BsSacB (3BYN)	BsSacB (6VHQ)	BsSacB (1PT2)	LjInuJ (2YFT)	LjInuJ (2YFS)
EtLsc (7OSO)		91.6%	47.2%	46.7%	29.3%	29.0%	29.3%	27.8%	27.5%
EaLsc (4D47)	91.6%		48.1%	47.5%	29.0%	28.7%	29.0%	28.1%	27.8%
ArFFase (3VSS)	47.2%	48.1%		64.1%	28.1%	27.8%	28.1%	24.9%	24.6%
BiBftA (6M0E)	46.7%	47.5%	64.1%		29.0%	28.7%	29.0%	26.4%	26.1%
BsSacB (3BYN)	29.3%	29.0%	28.1%	29.0%		99.7%	100.0%	43.5%	43.2%
BsSacB (6VHQ)	29.0%	28.7%	27.8%	28.7%	99.7%		99.7%	43.2%	43.2%
BsSacB (1PT2)	29.3%	29.0%	28.1%	29.0%	100.0%	99.7%		43.5%	43.2%
LjInuJ (2YFT)	27.8%	28.1%	24.9%	26.4%	43.5%	43.2%	43.5%		99.7%
LjInuJ (2YFS)	27.5%	27.8%	24.6%	26.1%	43.2%	43.2%	43.2%	99.7%	

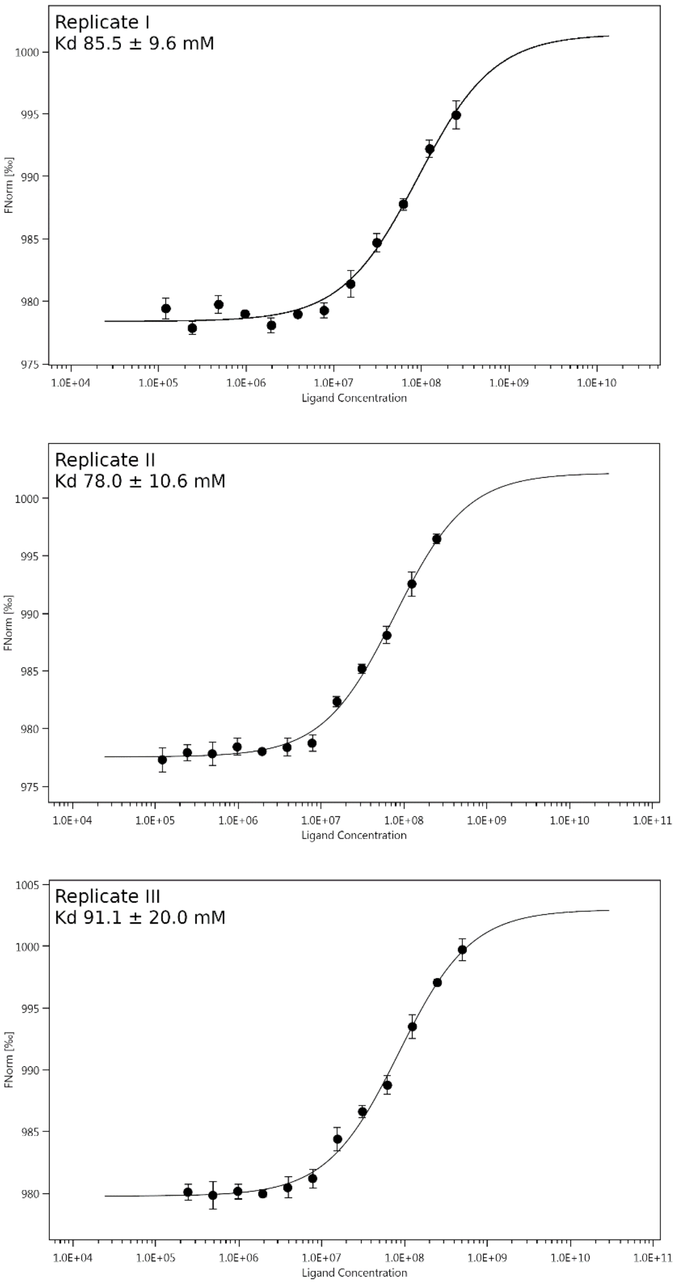

Figure S1 EtLsc MST binding assay. The data have been collected in replicate and processed with MO.Affinity Analysis from NanoTemper.

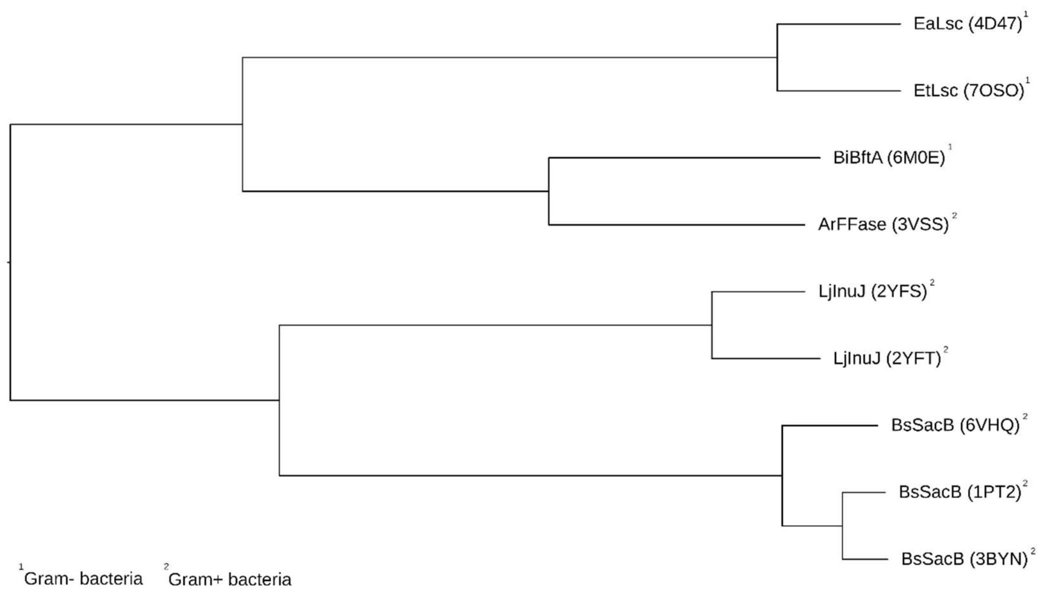

Figure S2 Structure identity comparison of bacterial LSCs and INUs. The tree was generated with DALI server and shows the Dali Z-scores. The structure similarity is represented by the branch length.

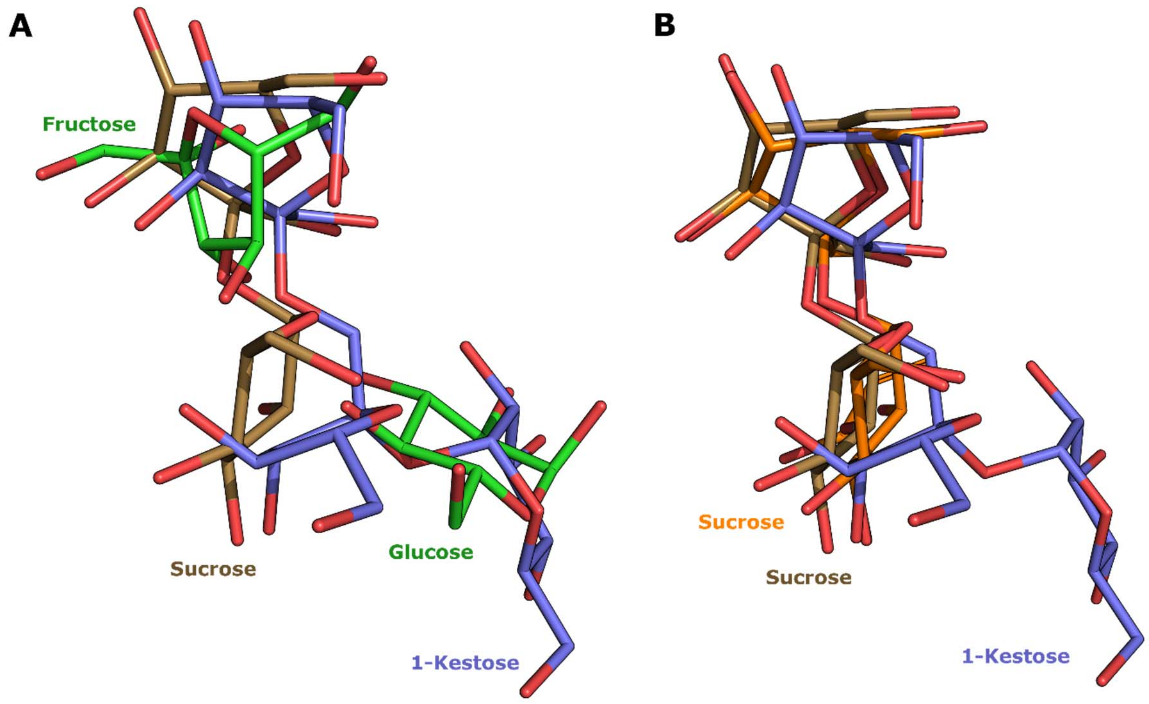

Figure S3 Fructose binding mode of InuHj from *Halalkalicoccus jeotgali B3T*. InuHj substrates 1-Kestose (PDB ID 7BJ4, coloured in *slate*) and sucrose (PDB ID 7BJC, coloured in *sand*) compared to: (**A**) fructose from EaLsc (in *green*) as representative of Gram-negative binding mode; (**B**) sucrose from BsSacB (in *orange*) as representative of Gram-positive binding mode.
